# General Remarks on Equine Ovariotomy

**Published:** 1894-03

**Authors:** James A. Waugh

**Affiliations:** Allegheny, Pa.


### GENERAL REMARKS ON EQUINE OVARIOTOMY.

By Jas. A. Waugh, V. S.,

It appears this important surgical operation has not yet received much attention or consideration from modern veterinarians in this country, as the most active thinkers and ablest workers in our profession are busily engaged in bacteriological investigations and researches, and in sanitary work in official capacities, while the money-making elements are generally engaged in lucrative practice according to the routine system as usually taught at our old style colleges. However, these are not valid excuses to retard the development and application of modern veterinary surgery.

Equine ovariotomy may be practiced as a matter of necessity or utility. We may resort to this operation for the correction and cure of a variety of diseased conditions of the female sexual system, which manifest themselves in a variety of ways, such as the obnoxious and disagreeable tail-switching and kicking habit, frequent hysteria, dysmenorrhœa, ovaritis, and as a prophylactic measure where mares are affected with maladie du coit or similar sexual diseases. Utility indicates this operation in case of valuable coach or fancy driving mares destined for matched teams and family use in large cities and towns, as it is well known that mares are not usually very saleable for these purposes, owing to their eccentric actions during their frequent menstrual periods. It would surely render mares more suitable and serviceable for general military purposes in all countries. Race mares would be saved from the inconvenience of their regular menstrual periods, which frequently interferes seriously with their racing qualities and money-earning capacities. Diseased and defective mares could be spayed, and thereby benefit the horse breeding interests in many ways, which would be very evident in the course of time, and quality would then command reasonable prices in all markets. The present lack of system and haphazard methods of breeding indiscriminately from all classes of mares, irrespective of condition, deteriorates values, and makes horses very uncertain property to purchase or own under existing circumstances, especially when bought without competent veterinary examination. Mare mules are improved and made more suitable and serviceable for all sorts of work, especially in hot climates, where the asinine and equine menstrual periods are more frequent and of longer duration than in the colder climates. A competent veterinary certificate of equine spaying would enhance the price and increase the value of any saleable mare mule or other mare undesirable for breeding and intended only for economic or racing purposes.

Prof. Liautard's "Animal Castration" contains excellent instructions and descriptions of this operation in mares, and it is deemed unnecessary to indulge in minute details in this article. However, I will cite a very interesting and successful case in my practice: A coal mining company called me to treat a sick mare mule, which I found suffering from fistulous withers and a very large and unopened abscess in the left gluteal region, due to fracture of the dorsal and sacral vertebra, which resulted from cruel beatings with spragging blocks in the hands of the mine mule drivers, when this mare mule indulged in very eccentric capers and refused to work during her menstrual periods. She was idle, sick and unfit for work, and I considered the case carefully, then recommended ovariotomy to remove the source and cause of the annoyance and trouble, and thereafter treat the fistula and abscess, which would soon recover under proper treatment, and the mule would then likely recover and soon be fit for work, same as any castrated mare mule. I returned to the city for special instruments necessary for this operation, and took my old friend, Dr. Rectenwald, out with me. We cast and secured the patient by the "Miles method," then I introduced my hand into the vagina and smeared the walls thoroughly with carbolized vaseline, passed the vaginal speculum to dilate and stretch the walls of the vagina, next introduced the hand armed with an old style embryotomy knife to make the necessary incision in the antero-superior wall of the vagina, and introduced the thumb and index finger through the wound, dilated it, and finally passed my hand, searched for and secured one ovary at a time. I found it impossible to introduce the ecraseur or any shears with the hand into the vagina, owing to its small size, and I pulled the ovaries, one at a time, to the surface external to the labia-majora, and used the ecrasuer for their removal. I then reapplied the antiseptic ointment to the walls of the vagina and returned the broad ligaments to their proper places,

The large abscess in gluteal region was opened and dressed antiseptically, then opened the fistula in the usual way on both sides, but removed a considerable flap at the top between the two openings to ensure free drainage and rapid recovery, dressed wounds antiseptically and left instructions and medicines for proper daily treatment. Result: complete and rapid recovery and restoration to usefulness at work in the mines.

This mule was quartered under unfavorable circumstances in an earth floor and frame shed, and received very little care and attention from its attendants, and blankets and bedding were not even used. Therefore, I believe spaying is not more dangerous than castration under similar circumstances.

ALLEGHENY, PA.

---

